# Supplementary material for: Nanoparticle-enabled phase control for arc welding of unweldable aluminum alloy 7075
Source: Nat Commun. 2019 Jan 9;10:98. doi: 10.1038/s41467-018-07989-y (PMC6327098; doi:10.1038/s41467-018-07989-y)
Supplement: Supplementary file 1 — Supplementary Information [file 41467_2018_7989_MOESM1_ESM.pdf]

Nanoparticle-enabled Phase Control for Arc Welding of Unweldable Aluminum Alloy 7075

Sokoluk et al.

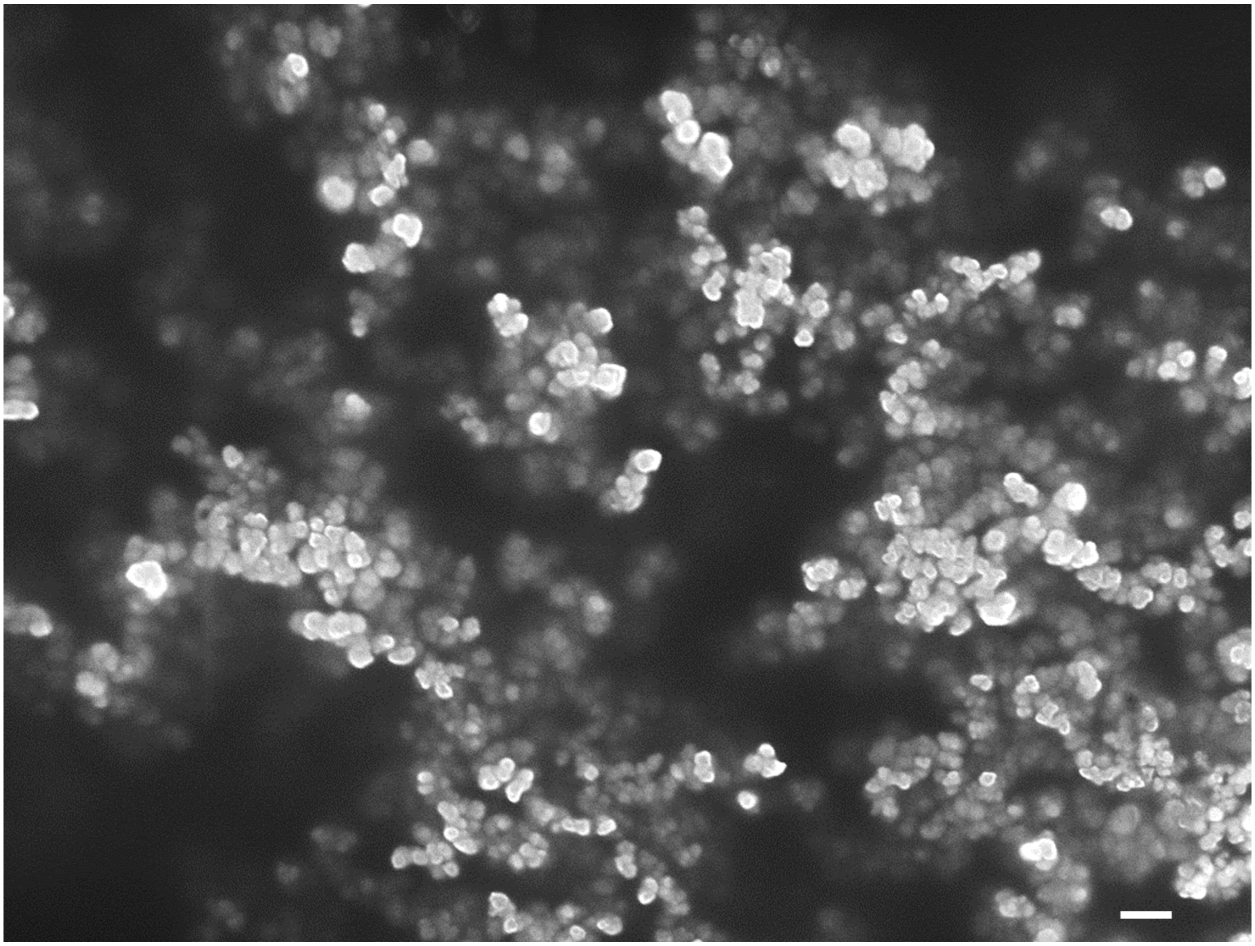

Supplementary Figure 1 | TiC nanoparticles as received. SEM image of the TiC nanoparticle powder as received from US Research Nanomaterials, Inc. Using flux assisted liquid state incorporation, these nanoparticles were incorporated into aluminum. Scale bar, 200 nm.

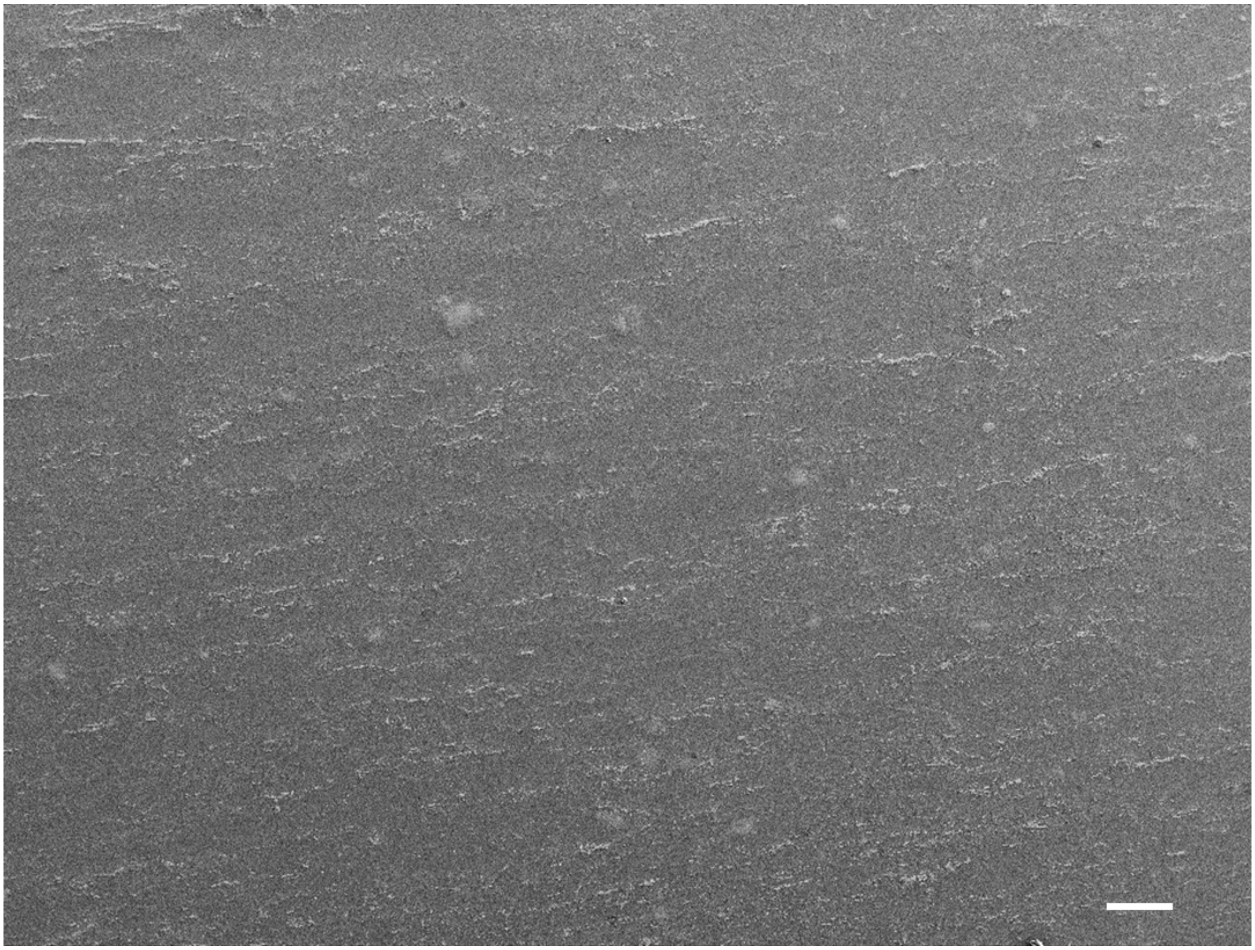

Supplementary Figure 2 | SEM Image of AA7075+TiC welding rod cross-section 1. The image shows TiC nanoparticle strips in extrusion direction. The occurrence of these strips is evenly distributed throughout the welding rod. Scale bar, 100 µm.

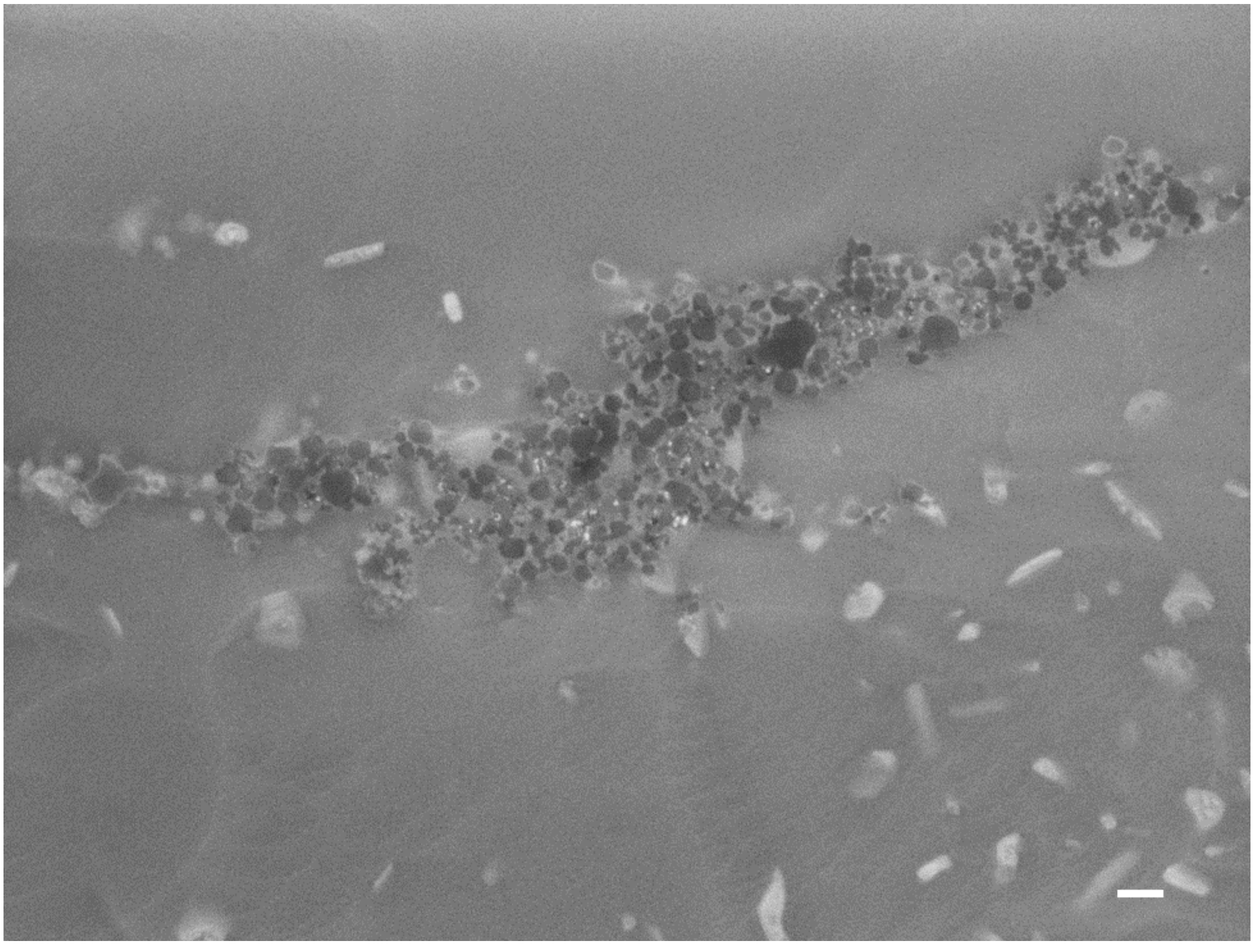

Supplementary Figure 3 | SEM Image of AA7075+TiC welding rod cross-section 2. High magnification image of one of the TiC particle (dark) strips shown in Supplementary Figure 2. TiC mainly remains within the AA7075's secondary phase at the grain boundaries. The nanoparticles appear to be deagglomerated and well dispersed within the secondary phase. Scale bar, 200nm
